# ArmTenna: Two-Armed RFID Explorer for Dynamic Warehouse Management

**DOI:** 10.3390/s26051513

**Published:** 2026-02-27

**Authors:** Abdussalam A. Alajami, Rafael Pous

**Affiliations:** Department of Information and Communication Technologies Engineering, Pompeu Fabra University, 08018 Barcelona, Spain

**Keywords:** RFID-based exploration robots, RFID-based navigation, indoor warehouse exploration, exploration robots, RFID-based robots, RFID-SLAM exploration

## Abstract

Efficient RFID spatial exploration in dynamic warehouse environments is challenging due to occlusions, sensing geometry constraints, and the weak coupling between information acquisition and navigation decisions. Many existing inventory robots treat RFID sensing as a passive data source during exploration, without explicitly optimizing sensing pose or prioritizing inventory-driven frontiers, which can result in incomplete coverage and redundant traversal. This paper presents ArmTenna, an articulated mobile robotic platform that formulates RFID inventory exploration as an active perception problem. The system integrates dual 4-DOF robotic arms carrying directional UHF RFID antennas and a 2-DOF neck-mounted RGB-D camera, enabling adaptive interrogation of candidate regions. We propose a multi-modal frontier exploration framework that combines newly detected EPC tags, average RSSI values, and vision-based product detections into a composite utility function for goal selection. By embedding articulated antenna control directly into the frontier evaluation loop, the robot tightly couples sensing geometry with exploration decisions. Experimental validation with 150 tagged items across three separated warehouse zones shows that ArmTenna achieves up to 97% map coverage, compared to 72% for a baseline platform, while reducing missed-tag regions. These results demonstrate that integrating active sensing pose control with multi-modal frontier evaluation provides an effective and scalable solution for RFID-driven warehouse inventory automation.

## 1. Introduction

### 1.1. Background

Efficient dynamic warehouse inventory management remains a fundamental challenge in modern logistics systems. Traditional inventory methods, often manual or semi-automated, frequently suffer from limited scalability, delayed updates, and incomplete coverage, which negatively affect supply chain performance and operational efficiency [1,2,3,4,5,6]. The increasing complexity of large-scale storage facilities and the demand for real-time inventory awareness have motivated the development of autonomous robotic solutions capable of performing systematic, repeatable, and reliable inventory operations. Autonomous inventory robots must simultaneously solve two tightly coupled problems: (i) spatial exploration and coverage of storage areas and (ii) reliable identification and localization of inventory items. Exploration is typically addressed through Simultaneous Localization and Mapping (SLAM) and frontier-based navigation strategies [7,8,9,10]. However, classical exploration frameworks were originally designed for geometric coverage of unknown environments rather than for inventory-driven sensing tasks. In warehouse scenarios, the objective is not merely to maximize spatial coverage, but to optimize inventory information gain under sensing constraints. Radio Frequency Identification (RFID) technology has emerged as a key enabler of automated inventory management due to its robustness, low cost, and ability to identify items without line-of-sight [11,12,13,14,15,16,17,18]. Integrating RFID sensing with autonomous mobile robots provides the potential for fully automated stock monitoring. Nevertheless, effective RFID-driven exploration requires intelligent coupling between navigation decisions and sensing performance, especially in cluttered warehouse environments where occlusions, shelving geometry, and multipath effects significantly influence tag detection reliability.

### 1.2. Related Work

Autonomous exploration has been extensively studied in robotics, including frontier-based exploration [8,9,19], information-theoretic and utility-based methods [20], metaheuristic strategies [21], deep reinforcement learning approaches [22], and clustering-based techniques [23]. While these methods enable efficient environment coverage, many assume predefined exploration boundaries or prioritize geometric map completion rather than task-driven information acquisition. In inventory contexts, unconstrained exploration can lead to inefficient traversal of irrelevant regions. RFID-enhanced robotic systems have been investigated for localization [11,12], trajectory tracking [13], navigation [14,15], obstacle avoidance [16], and autonomous exploration [17,18]. Polvara et al. [14] proposed a multi-criteria coverage strategy for RFID-based navigation under sparse tag conditions, but assumed availability of a known map, limiting applicability to dynamic warehouse environments. Stigmergic RFID navigation [6,18,24] introduced gradient-based movement toward newly detected tags, enabling lightweight decentralized coordination. However, such approaches do not guarantee systematic RFID-space coverage and typically lack explicit stopping conditions or coverage guarantees. Recent works have incorporated mapping and optimization techniques to improve RFID inventory efficiency. Multi-modal mapping systems combining LiDAR and RGB-D sensing with route optimization methods such as Ant Colony Optimization (ACO) have demonstrated improved traversal efficiency [25]. The Next-Best-Sense (NBS) framework [14] addressed RFID localization accuracy by selecting sensing poses that maximize expected tag information gain. Similarly, Next-Best-View (NBV) strategies in robotic perception emphasize viewpoint optimization for improved sensing quality. However, most RFID exploration systems rely on fixed antenna configurations, which constrain directional interrogation and reduce the ability to associate detected tags with specific spatial frontiers in cluttered warehouse geometries. More recently, RFID frontier-based exploration [26] introduced navigation toward frontiers associated with high tag densities. While promising, the fixed antenna setup limited precise spatial discrimination between adjacent frontiers, especially in multi-aisle or occluded shelf configurations. Across the literature, a common limitation emerges: existing systems treat RFID sensing as a passive modality attached to a mobile base, rather than as an actively controllable sensing mechanism. Consequently, viewpoint control, directional interrogation, and adaptive sensing pose optimization remain underexplored in warehouse-scale RFID robotics. Recent trends toward active sensing and multi-modal inventory robots have been observed. Recent research increasingly recognizes that RFID inventory performance is strongly dependent on sensing geometry and platform motion. For example, rotating or moving antennas have been proposed to improve inventory performance and enable more reliable localization in dense shelving systems [27]. Similarly, vision-guided dynamic antenna positioning has been shown to substantially improve detection rates compared to static configurations in cluttered, multi-tier shelves [28]. Multi-modal fusion between RFID and vision has also been explored to mitigate ambiguity in high-density tag environments and improve the robustness of inventory identification [5]. In parallel, emerging mobile platforms (including legged robots) are being investigated to enable inventory monitoring in environments where navigation constraints limit conventional wheeled systems [29]. These directions collectively motivate the need for integrated robotic systems that combine active, controllable RFID sensing with multi-modal exploration policies, which is the focus of this work.

Despite significant progress in RFID-enabled mobile robotics, existing systems predominantly rely on fixed-antenna configurations and treat RFID sensing as a passive modality coupled to base motion. As a result, sensing geometry is not explicitly optimized, and detected tags cannot always be reliably associated with individual frontiers in cluttered warehouse layouts characterized by shelving occlusions and multipath effects. Moreover, exploration strategies often prioritize geometric map completion rather than inventory-driven information gain. This work addresses these limitations by explicitly formulating RFID inventory exploration as an active perception problem, in which sensing pose, sensing direction, and navigation decisions are jointly optimized. The main contributions of this paper are:

1. An articulated RFID exploration robot enabling active sensing pose control: We present a mobile robotic platform equipped with dual four-DOF articulated arms carrying directional RFID antennas and a two-DOF neck-mounted RGB-D sensor. Unlike fixed-antenna systems, the proposed platform enables deliberate and adaptive interrogation of candidate frontiers through controlled antenna orientation, improving tag acquisition in occluded and high-density shelving environments.
2. A multi-modal frontier utility formulation for task-driven exploration: We introduce a unified frontier evaluation framework that jointly reasons over (i) newly observed unique EPCs, (ii) RSSI statistics reflecting signal confidence and proximity, and (iii) visually detected product-like objects. This formulation shifts exploration from purely geometric coverage toward inventory-relevant information gain.
3. Integration of articulated sensing into the frontier-based exploration loop: We embed active antenna interrogation into the decision-making cycle such that each frontier candidate is evaluated through deliberate multi-sensor observation before navigation commitment. This enables improved spatial attribution of RFID observations and reduces exploration redundancy in structured warehouse layouts.
4. Quantitative experimental validation against a fixed-antenna baseline: Through controlled multi-zone warehouse experiments, we demonstrate that articulated sensing combined with multi-modal fusion improves inventory coverage and tag discovery performance compared to a conventional fixed-antenna RFID robot.

The proposed system advances RFID-enabled warehouse robotics beyond passive sensing paradigms and establishes a scalable framework for high-density, cluttered inventory environments.

## 2. Robots Used

### 2.1. Hardware Description

#### 2.1.1. ArmTenna Robot

**Figure 1:**
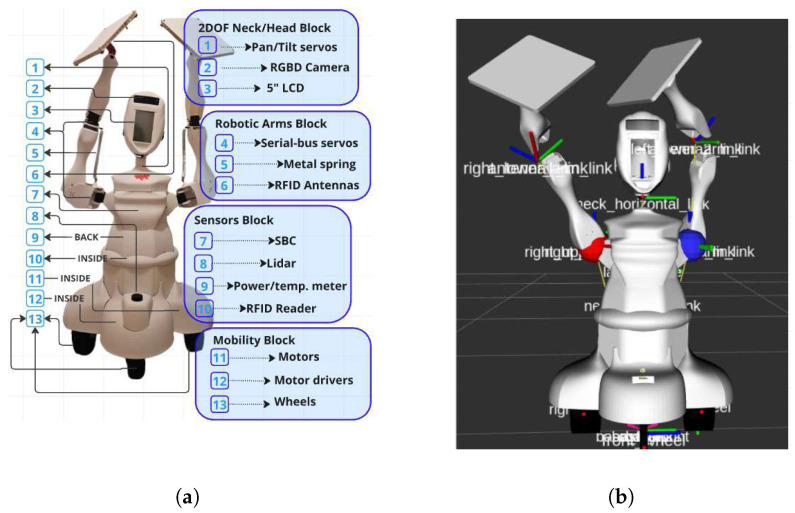
ArmTennahardware and RViz description: (**a**) Hardware description of the ArmTenna robot; (**b**) illustration of the RViz representation.

The robot hardware, as shown in Figure 1, can be explained in four blocks, as follows:

1. Two-DOF neck/head Block
  (a) Pan-Tilt servos: two-DOF actuators are used enabling precise head movement in both horizontal (pan) and vertical (tilt) directions, allowing for targeted visual inspection of warehouse environments.
  (b) RGBD Camera: An AI-enabled depth-sensing camera that captures both RGB color images and depth information is used, enabling 3D perception and object detection capabilities.
  (c) Display screen: A 5-inch touchscreen LCD display used for real-time visual feedback and system status monitoring and control is mounted that resembles the face of the robot.
2. Robotic Arms Block
  (a) Serial-bus servos: High-precision actuators are connected via serial communication, providing controlled movement for each of the four DOFs in both robotic arms. These servos enable accurate positioning of RFID antennas for inventory scanning.
  (b) Metal spring: Mechanical dampening elements that provide stability and shock absorption during arm movements.
  (c) RFID antenna (Advantenna-SP11): A compact RFID UHF antenna with very high gain and circular polarization. This antenna features a wide beam radiation pattern in all directions within one hemisphere, making it ideal for the ArmTenna application.
3. Sensors Block
  (a) Single-board computer (SBC): An n100 processor, 16gb RAM SBC serves as the robot’s central processing unit, handling computations for navigation, object detection, and RFID data processing.
  (b) 2D Lidar: The Lidar used is a 7 m Ranging sensor that is used to create detailed 2D maps of the environment by measuring distances to obstacles, essential for navigation and frontier detection.
  (c) Power/temp. meter: A monitoring system is installed that tracks power consumption and temperature levels to ensure optimal performance and prevent system overheating.
  (d) RFID reader (AdvanReader-160): A high-power (31.5 dBm) four-port UHF RFID reader with an on-board microcomputer and a fully open Linux operating system.
4. Mobility Block
  (a) Motors: The robot has two 24v DC Motors providing propulsion power for the robot’s movement through warehouse environments.
  (b) Motor drivers: Electronic control units are connected to the motors to regulate the power delivery to the motors, enabling precise speed and direction control.
  (c) Wheels: Four 3D-printed thermoplastic polyurethane (TPU) wheels designed for stable movement on warehouse floors, supporting the robot’s weight. Two lateral wheels are connected to the motors and two smaller omnidirectional wheels are placed on the front and back for stability.

#### 2.1.2. Robin50

**Figure 2:**
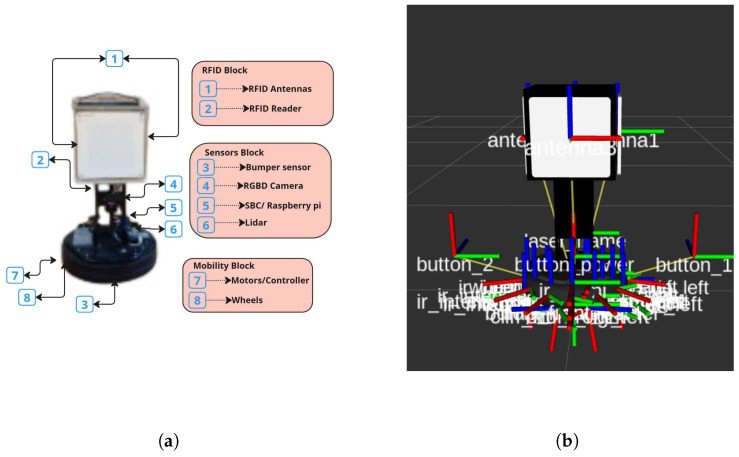
Robin50 hardware and RViz description. (**a**) Hardware description of the Robin50 robot; (**b**) illustration of the RViz representation.

The hardware description of the Robin50 robot shown in Figure 2a,b can be described in three parts:

1. Sensors Block
  (a) SBC: A Raspberry Pi serves as the central processing unit, managing sensor data integration, decision-making algorithms, and overall system control.
  (b) 2D Lidar: This sensor creates planar distance measurements, essential for obstacle detection, mapping, and navigation in dynamic environments.
  (c) RGB-D camera: An Intel D400 camera provides both color and depth information, enabling advanced perception tasks such as object recognition and 3D mapping.
  (d) Bumper sensors: These tactile sensors detect physical contact with obstacles, providing an additional layer of collision prevention.
2. Mobility Block
  (a) Base platform: The iCreate3 robot base forms the foundation of Robin50’s mobility system, offering differential drive movement for precise maneuverability and rotation in place.
  (b) Wheel encoders: Integrated encoders provide odometry data for accurate position estimation and dead reckoning.
  (c) Cliff sensors: These downward-facing sensors prevent the robot from falling off edges or stairs.

### 2.2. Software Description

#### 2.2.1. ArmTenna Robot

**Figure 3:**
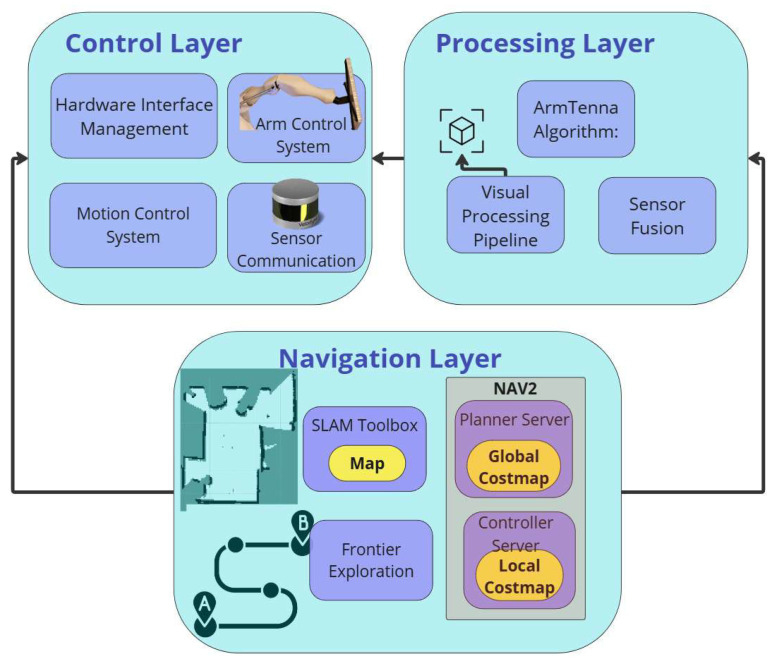
An illustration of the the robot’s software architecture with three layers: the control layer, processing layer, and navigation layer.

The robot software architecture implements a hierarchical three-layer system, as shown in Figure 3, with each layer specifically designed to manage different aspects of the robot’s operation while maintaining seamless integration between hardware components and algorithmic processes.

1. Control Layer
  (a) Hardware interface management: Implements direct communication with Dynamixel servo networks through the RS-485 protocol [30], managing both the two-DOF neck mechanism and dual four-DOF robotic arms at precise control frequencies.
  (b) Motion control system: The coordination of the differential drive system through dedicated motor drivers is done through ROS2-Control software [31], implementing real-time velocity and position control of the two-wheeled base configuration with two caster wheels for stability. This setup allows for efficient linear and rotational movements, enabling precise navigation in warehouse environments.
  (c) Sensor communication: An ROS2 node that manages data acquisition from the RGBD camera, LIDAR, and RFID reader systems, ensuring synchronized data collection and transmission.
  (d) Arm Control System: An ROS2 node that, given a target 3D position, implements inverse kinematics servo angle calculations for precise antenna positioning towards that position, utilizing the robotic arms to optimize RFID tag detection coverage.
2. Processing Layer
  (a) ArmTenna algorithm: This algorithm processes RFID tag data collected through antenna-equipped end effectors, analyzing signal strength and tag density distributions to optimize exploration strategies and inventory management. Details on this algorithm will be explained in Section 3.
  (b) Visual processing pipeline: Utilizes the onboard SBC to run YOLOv8 [32] object detection, processing RGBD camera feeds to identify Product-like objects (POs) within the warehouse environment. The YOLOv8 model was specifically trained on a dataset of 3000 images/classes containing various types of boxes, shelves, and common warehouse storage units. This tailored training enables the model to accurately detect and classify objects typically found in warehouse settings. The detection of these objects serves as a valuable hint for the ArmTenna robot, indicating potential locations where RFID tags might be present in the environment.
  (c) System integration: Maintains continuous communication between all three layers through ROS2 Humble middle-ware, ensuring coordinated operation of all hardware components for efficient warehouse exploration and inventory management.
  (d) Sensor Fusion: Integrates feedback from all hardware components, including power/temperature monitoring, to maintain optimal system performance and safety parameters.
3. Navigation Layer
  (a) SLAM implementation [33]: Processes LIDAR scans and odometry data to construct and maintain accurate environmental maps, enabling reliable robot localization within the warehouse space.
  (b) Path planning Framework (NAV2) [34]: Utilizes Nav2’s costmap generation and path planning algorithms to ensure efficient obstacle avoidance and smooth trajectory execution through the wheels.
  (c) Frontier exploration [10]: Implements advanced frontier detection algorithms that combine traditional exploration strategies with RFID and visual data to optimize warehouse coverage patterns.

The robotic platform was custom-designed and fully integrated as a unified system architecture that combines commercial sensing and actuation components within a cohesive hardware–software framework. Beyond system integration, the articulated antenna control strategy and the multi-modal frontier evaluation algorithm constitute original developments of this work, enabling active sensing pose control and task-driven exploration.

#### 2.2.2. Robin50

The software architecture of Robin50 presents a more simple, streamlined design compared to its predecessor, the ArmTenna robot, due to the absence of robotic arms and AI models. However, the navigation and obstacle detection stack remains largely similar. Robin50’s exploration strategy leverages its RFID sensing capabilities to guide its movement through the environment.

Robin50 employs an RFID-based exploration technique. The robot is equipped with four RFID antennas, strategically positioned as illustrated in Figure 2a,b. These antennas continuously scan the environment for unique RFID tags. The robot has an exploration algorithm which only processes this data to determine the most promising direction for exploration. Further details are found in [18].

## 3. System Architecture and Operation Flow

**Figure 4:**
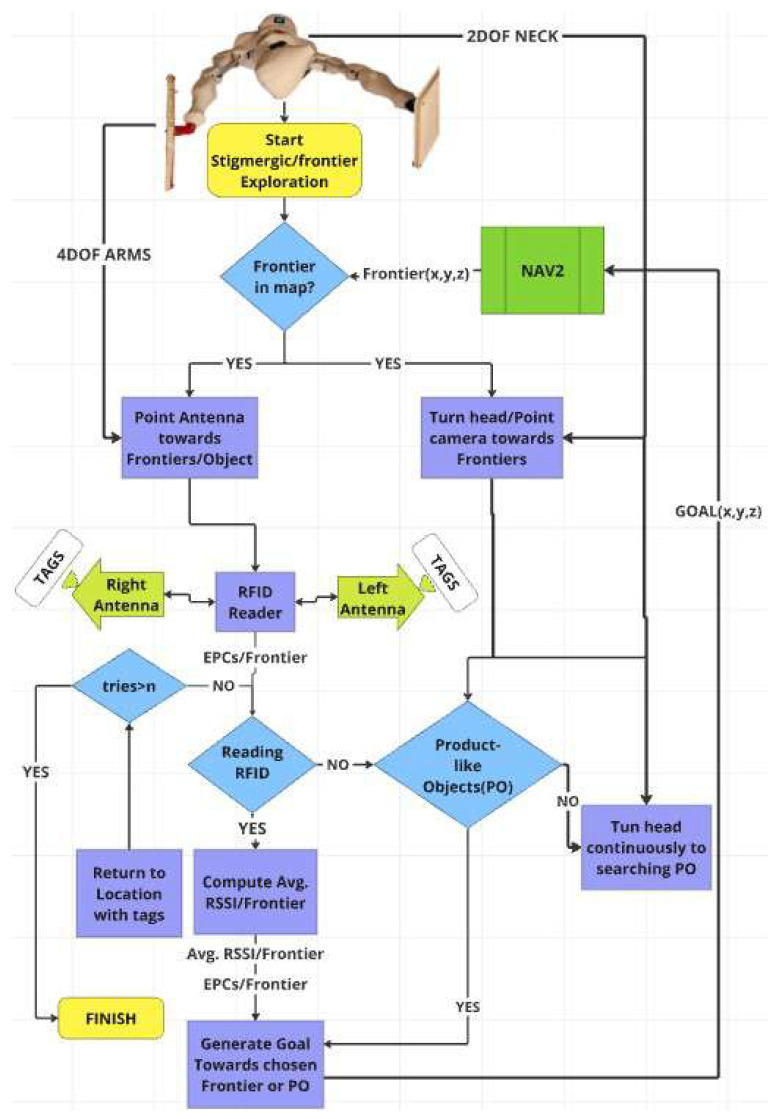
The block diagram of the operation flow of the ArmTenna robot.

The RFID reader in the ArmTenna system emits electromagnetic waves through its antennas, which are strategically positioned on the robot’s articulated arms. When these waves encounter RFID tags in the environment, they induce a small current in the tags’ antennas. This current powers the tags’ circuits, enabling them to transmit their stored information back to the reader. The reader then processes this returned signal, decoding the unique identifier and any additional data stored on the tag, which is subsequently used by the robot’s decision-making algorithms to guide its exploration and inventory management tasks. Furthermore, the warehouse exploration system begins with exploring the map for new possible frontiers using a frontier-based exploration strategy where frontiers are defined as boundaries between known and unknown spaces in the occupancy grid or so-called map, which interfaces directly with the ROS2 Nav2 stack, as illustrated in the operation flow block diagram in Figure 4. This architecture enables robust navigation and localization while maintaining a dynamic occupancy grid map. Let F be the set of all detected frontiers, as shown in Equation (1):

(1) $$F=F_{1},F_{2},...,F_{n}$$

(2) $$F_{i}=\left(x_{i},y_{i}\right)\;\mathrm{for}\;i\in 1,...,n$$

where $F_{i}$ in Equation (2) represents the i-th frontier with its 2D coordinates $\left(x_{i},y_{i}\right)$ on the 2D map.

After the frontier exploration step, the robot employs a dual-sensing approach, as follows:

1. RFID sensing: Two four-DOF robotic arms equipped with directional RFID antennas perform systematic scanning of identified frontiers by moving the arms to direct the antennas towards each frontier in range. The RFID reader processes signals from both left and right antennas, calculating unique RFID tags or so-called electronic product codes (EPCs) per frontier and average received signal strength indicator (RSSI) values from all the newly detected unique tags.
2. Visual detection: A two-DOF neck mechanism controls an RGBD camera, enabling simultaneous visual inspection of frontiers. This system actively searches for product-like objects (POs) through a pre-trained YOLO8 model using computer vision algorithms while the RFID scanning occurs.

The following step in the system implements a hierarchical decision-making framework. When a frontier is detected, both sensing systems (RFID and visual) are activated simultaneously. The RFID subsystem attempts readings with a maximum number of attempts (n), returning to successful detection locations if EPCs are detected. The visual system continuously monitors for POs. Finally, data from both systems is integrated to generate optimum navigation goals towards high-density RFID regions/frontiers. This is done based on three key metrics:

1. The new and unique EPCs per frontier $F_{i}$, which is defined as $n_{tags}\left(F_{i}\right)$.
2. The average RSSI values for the EPCs detected per frontier $F_{i}$, which is defined as $r_{avg}\left(F_{i}\right)$.
3. The number of visually detected POs per frontier $F_{i}$, which is defined as $o_{det}\left(F_{i}\right)$.

These parameters are weighted and combined to determine optimal exploration targets, ensuring comprehensive coverage while prioritizing areas with higher inventory presence.

For each frontier $F_{i}$, we define a composite score $S(F_{i})$ as follows:

(3) $$S\left(F_{i}\right)=\alpha \cdot N_{tags}\left(F_{i}\right)+\beta \cdot R_{avg}\left(F_{i}\right)+\gamma \cdot O_{det}\left(F_{i}\right)\;$$

where

$N_{tags}\left(F_{i}\right)$ is the normalized number of unique EPCs detected at each frontier.

$R_{avg}\left(F_{i}\right)$ is the normalized average RSSI value of all new unique detected tags at a frontier position.

$O_{det}\left(F_{i}\right)$ is the normalized number of POs detected at each frontier.

α, β, and γ are weighting coefficients, where α+β+γ=1.

The normalization for each parameter is calculated as follows:

(4) $$N_{tags}\left(F_{i}\right)=\frac{n_{tags}\left(F_{i}\right)}{\max_{j}\left(n_{tags}\left(F_{j}\right)\right)}$$

(5) $$R_{avg}\left(F_{i}\right)=\frac{r_{avg}\left(F_{i}\right)-r_{min}}{r_{max}-r_{min}}$$

(6) $$O_{det}\left(F_{i}\right)=\frac{o_{det}\left(F_{i}\right)}{\max_{j}\left(o_{det}\left(F_{j}\right)\right)}$$

The optimal frontier $F_{opt}$ is selected as:

(7) $$F_{opt}=argmax_{F_{i}}S\left(F_{i}\right)\;$$

where

$n_{tags}\left(F_{i}\right)$ is the number of tags detected at $F_{i}$, and the denominator is the maximum number of tags detected at any frontier.

$r_{avg}\left(F_{i}\right)$ is the average RSSI at $F_{i}$, and $r_{min}$ and $r_{max}$ represent the minimum and maximum RSSI values across all frontiers.

$o_{det}\left(F_{i}\right)$ is calculated at $F_{i}$, normalized by the maximum number of POs detected at any frontier.

The frontier with the highest composite score $F_{opt}$ is selected, effectively choosing the most promising area for further exploration.

The weighting coefficients can be dynamically adjusted based on the warehouse environment and inventory management priorities. The values used for conducting the experiments in this paper are as follows:

α=0.4 (EPC count priority).

β = 0.3 (signal strength consideration).

γ = 0.3 (visual detection weight).

The weighting coefficients α, β, and γ are selected to balance inventory-driven information gain and sensing reliability. A higher weight (α = 0.4) is assigned to the normalized number of newly detected unique EPCs, as maximizing new tag discovery is the primary objective of the exploration task. The remaining weights (β = 0.3 for RSSI and γ = 0.3 for visual detections) ensure that signal confidence and product-like object evidence contribute to frontier prioritization without dominating the decision process. These values were determined empirically through preliminary experiments to achieve stable and consistent exploration behavior across different warehouse layouts. The framework remains modular, and the weights can be adjusted according to operational priorities or environment characteristics. To ensure safe operation during antenna articulation, arm motions are constrained by predefined joint limits and workspace boundaries derived from the robot kinematic model. Antenna pointing is performed only after the navigation stack positions the mobile base at a safe clearance from shelves or walls. The interrogation envelope is restricted to remain within a bounded region relative to the base footprint, and arm velocities are limited to reduce impact risk. These constraints prevent collisions during directional RFID interrogation in structured warehouse environments.

The proposed exploration framework is robot-agnostic at the algorithmic level. It requires only (i) a navigation stack capable of generating frontier candidates and executing waypoint goals and (ii) an RFID sensing subsystem providing EPC and RSSI statistics (optionally complemented by vision-based detections), independent of a specific robot morphology.

## 4. Experiments

**Figure 5:**
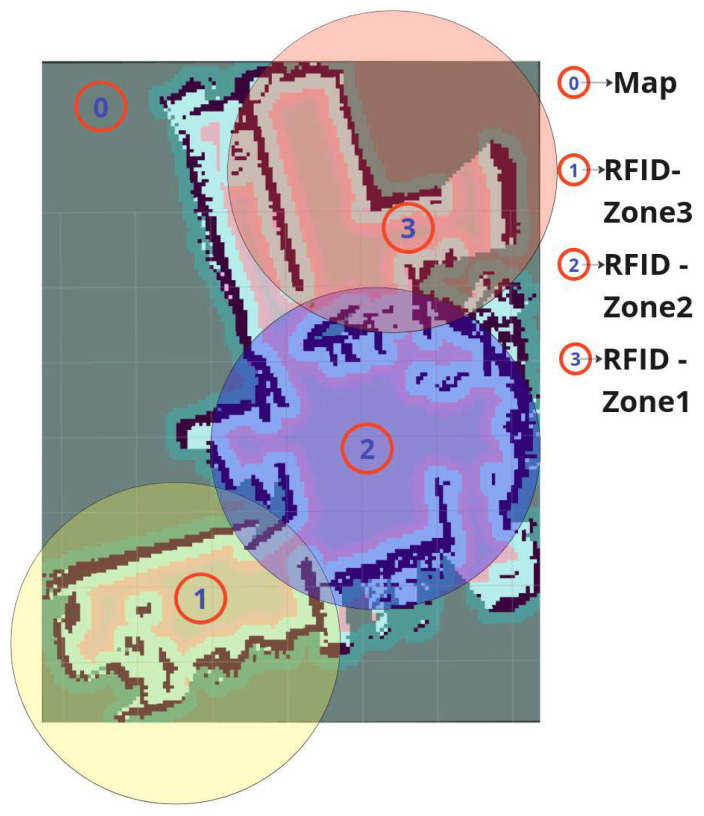
An illustrationof the Map and RFID zones of the laboratory.

To evaluate the effectiveness of different RFID-based exploration strategies, we conducted experiments comparing the Robin50 and ArmTenna robots in a warehouse environment with total 150 RFID tags divided into three distinct RFID tag zones, as shown in Figure 5. The experiments aimed to assess each robot’s ability to explore and detect RFID tags across these zones. To enable a clear and reproducible comparison between the articulated platform and the fixed-antenna baseline, the evaluation focuses on quantitative metrics directly derived from logged exploration data. Specifically, we measure (i) RFID discovery coverage, defined as the percentage of deployed EPC tags detected at least once during the mission; (ii) environment coverage, computed from the explored occupancy grid relative to the accessible area; and (iii) exploration efficiency, expressed in terms of traveled distance and mission duration. These metrics jointly characterize both inventory sensing performance and navigation behavior.

The warehouse environment was divided into three zones:

1. Zone 1: The initial starting area marked in yellow in Figure 5, consists of 50 RFID tags.
2. Zone 2: Adjacent to Zone 1 and marked in blue in Figure 5, separated by a partial wall and consisting of 50 RFID tags.
3. Zone 3: Separated from Zone 2 by a full wall and marked as red in Figure 5, accessible only through a specific path and consisting of 50 RFID tags.

**Figure 6:**
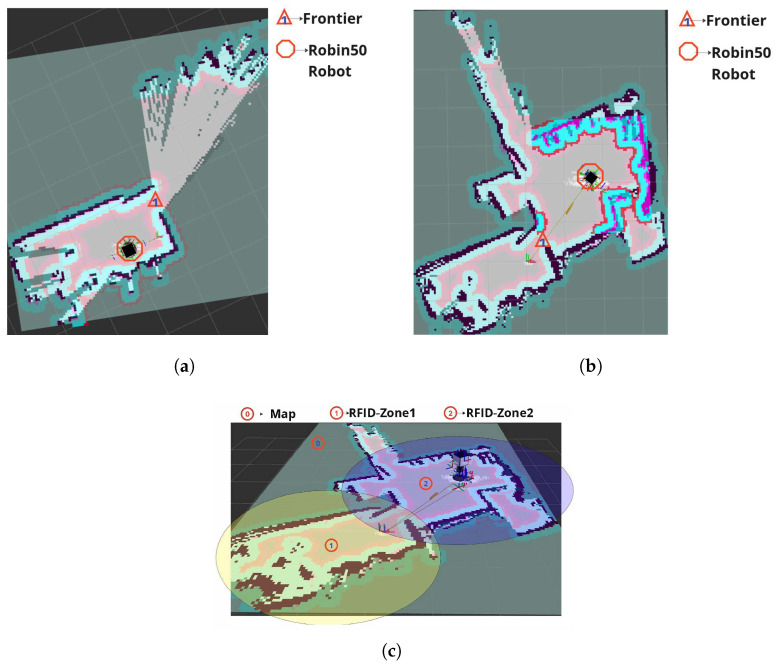
Robin50 exploration phases: (**a**) Robin50 initial starting area in Zone 1; (**b**) Robin50 successfully exploring Zone 2; (**c**) Robin50 finished its exploration mission and remained in Zone 2.

Both robots started their exploration from the same initial position in Zone 1, as shown in Figure 6a.

### 4.1. Experiment 1: Robin50 Exploration

The Robin50 robot employed its RFID-guided exploration strategy, which relies on analyzing the number of unique RFID tags detected by each of its four antennas during predefined time intervals. The robot’s navigation was determined by selecting the direction corresponding to the antenna that detected the highest number of unique tags and setting a goal with a given distance (1 m in this experiment) in that direction. The results of Robin50 can be concluded as follows:

1. It successfully explored Zone 1, as shown in Figure 6a.
2. It transitioned to and explored Zone 2, as shown in Figure 6b.
3. It failed to reach Zone 3 and finished its mission with an exploration of approximately 72% of the entire map, as shown in Figure 6c.

Robin50’s strategy led it to continuously navigate within Zones 1 and 2, following the direction of detected tags. However, this approach proved limiting, as the robot was unable to discover the path to Zone 3 due to the intervening wall and because its exploration strategy did not guide it towards the specific gap or path that leads to Zone 3. The robot remained in Zone 2, repeatedly scanning until no new tags were detected, ultimately failing to explore the entire environment.

### 4.2. Experiment 2: ArmTenna Exploration

The ArmTenna robot utilized its newly developed two-armed RFID exploration method. First, the robot started to detect the frontiers. The robot identified unexplored areas (frontiers) on its map. Second, it performed targeted sensing. Using its articulated arms, the robot pointed RFID antennas towards different frontiers and started reading RFID tags. Finally, it performed multi-factor analysis. For each frontier, the robot measured the total number of RFID tags detected, the average RSSI of new tags, and the number of POs detected by its YOLO model, as explained in Section 3.

**Figure 7:**
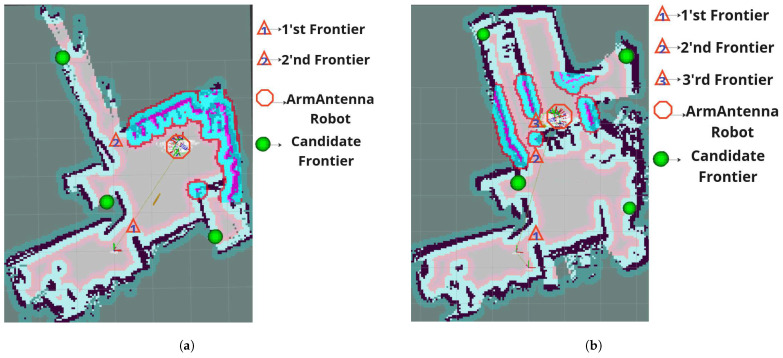
ArmTenna exploration phases. (**a**) ArmTenna successfully exploring Zone 1 and 2; (**b**) ArmTenna successfully exploring Zone 3.

The results of the ArmTenna robot can be concluded as follows:

1. It efficiently explored Zone 1, as shown in Figure 7a.
2. It quickly transitioned to and explored Zone 2 while reading all the EPCs in the zone, as shown in Figure 7a.
3. It successfully discovered and explored Zone 3 with an exploration of approximately 97% of the entire map, as shown in Figure 7b.

ArmTenna’s decision-making process allowed it to identify and prioritize frontiers that led to unexplored areas. This approach enabled the robot to discover the path to Zone 3, despite the presence of walls and obstacles. The ArmTenna robot demonstrated superior exploration capabilities compared to Robin50:

1. Speed: ArmTenna reached Zone 2 quicker than Robin50.
2. Completeness: While Robin50 was confined to Zones 1 and 2 and only explored 72% of the map, ArmTenna successfully explored all three zones, covering approximately 97% of the map.
3. Efficiency: ArmTenna’s multi-factor analysis allowed for more informed decision-making, leading to a more thorough and efficient exploration pattern.

These experiments highlight the advantages of ArmTenna’s advanced exploration strategy, which combines frontier-based exploration with multi-modal sensing. This approach proves to be more effective in complex environments with obstacles and separated zones, providing a more comprehensive coverage of RFID-tagged inventory areas. Although the experimental layout was kept structurally static during each run to enable controlled comparison between platforms, the proposed framework is designed for dynamic warehouse environments. The SLAM-based navigation stack continuously updates the occupancy map, and frontier generation is recomputed online, allowing adaptation to moving obstacles or layout changes. Future work will extend the experimental validation to scenarios involving dynamic obstacles, shelf reconfiguration, and real-time tag redistribution to further assess robustness under realistic warehouse conditions.

## 5. Future Work

While our current results are promising, there are several avenues for future research and development:

1. Enhanced Object Recognition: Improving the neural network’s ability to recognize a wider range of products and packaging types could further enhance the robot’s inventory management capabilities.
2. Multi-Robot Coordination: Investigating the potential for multiple robots to work collaboratively could significantly increase the efficiency of large-scale warehouse exploration and inventory management.
3. Integration with Warehouse Management Systems: Developing seamless integration with existing warehouse management systems could provide real-time inventory updates and optimize overall logistics operations.
4. Adaptive Exploration Strategies: Implementing machine learning algorithms to adapt the robot’s exploration strategy based on historical data and changing warehouse conditions could further improve efficiency.
5. Human–Robot Collaboration: Exploring ways for the robot to work alongside human warehouse staff could lead to more flexible and efficient inventory management processes.

These future directions aim to build upon the foundation laid by our current work, further advancing the field of automated warehouse inventory management.

## 6. Conclusions

The ArmTenna robot represents a promising solution for significant advancements in automated warehouse inventory management through its integration of articulated RFID sensing, computer vision, and advanced exploration strategies. Our experimental results demonstrate that the combination of dual four-DOF robotic arms for targeted RFID scanning and a two-DOF neck-mounted camera for visual object detection creates a highly effective solution for comprehensive inventory tracking. The system’s multi-modal approach, leveraging weighted parameters of RFID tag density, signal strength measurements, and visual object detection, enables more intelligent and efficient exploration patterns compared to traditional fixed-antenna configurations. This sophisticated decision-making framework allows the robot to dynamically adapt its exploration strategy, ensuring thorough coverage while prioritizing areas with higher inventory concentrations. Through rigorous experimental validation, ArmTenna has demonstrated its superior capabilities, successfully exploring approximately 97% of the RFID-tagged environment, a stark contrast to Robin50’s 72% coverage. This marked improvement underscores the effectiveness of ArmTenna’s articulated design and multi-modal sensing approach. This dual-sensing capability, combined with the robot’s articulated design, addresses the critical limitations of existing solutions, particularly in accessing and identifying inventory in complex warehouse layouts. While further research and real-world testing are necessary to fully validate these findings, our results articulate improvements in coverage efficiency, tag detection accuracy, and inventory comprehensiveness that suggest that this approach could enhance the scalability and reliability of automated warehouse operations.

## Data Availability

The raw data supporting the conclusions of this article will be made available by the authors on request.
